# The protective effect of Ganoderma lucidum on testicular torsion/detorsion-induced ischemia-reperfusion (I/R) injury ^1^

**DOI:** 10.1590/s0102-865020200010000003

**Published:** 2020-03-20

**Authors:** Gül Doğan, Hülya İpek

**Affiliations:** I MD, Hitit University Faculty of Medicine , Department of Pediatric Surgery , Çorum , Turkey . Acquisition of data, manuscript writing.; II MD, Hitit University Erol Olcok Training and Research Hospital , Department of Pediatric Surgery , Çorum , Turkey . Acquisition of data, manuscript preparation.

**Keywords:** Spermatic Cord Torsion, Testis, Reishi, Vascular Endothelial Growth Factor A, Lymphoma, B-Cell, Rats

## Abstract

**Purpose:**

To investigate the protective effect of *Ganoderma lucidum* on testicular torsion/detorsion (T/D)-induced ischemia-reperfusion (I/R) injury.

**Methods:**

Thirty male Wistar albino rats were randomly categorized into 3 groups: Group 1: sham, Group 2 ( T/D): 2,5 hours of ischemia and 7 days of reperfusion, Group 3 (T/D+ *G. lucidum* ): 2,5 hours of ischemia and 7 days of reperfusion and 7 days of 20 mg/kg via gastric gavage *G. lucidum* polysaccharides per day. Biochemical assays of Malondialdehyde (MDA), superoxide dismutase (SOD), Catalase (CAT), Glutathione (GSH) levels , histopathology and expression levels of VEGF and Bcl-2 with immunohistochemical methods were examined in testicular tissue.

**Results:**

*G. lucidum* treatment was found to have prevented the T/D-induced I/R injury by decreasing MDA levels of the testis. SOD, CAT and GSH activities were decreased in group 2, while they were increased in group 3 (p<0.001) and significant improvement in the tube diameter was observed in group 3. Bcl-2-positive germinal cells were lowered in group 3 compared to the group 2. VEGF expression showed an increase in group 2, whereas it decreased in group 3.

**Conclusion:**

The antioxidant *G. lucidum* is thought to induce angiogenesis by reducing the apoptotic effect in testicular torsion-detorsion.

## Introduction

Testicular torsion is among the most common urological emergencies observed in children. The annual incidence of spermatic cord torsion is 4.5 in 100,000 males aged 1-25 years ^1^ . This condition may cause ischemia and loss of the testis. Testicular damage varies depending on the grade and duration of the torsion ^2^ . Testicular torsion decreases blood flow to the testis, causing tissue ischemia. The accumulation of reactive oxygen species (ROS) is the underlying pathologic mechanism of testicular torsion followed by ischemia, and it has been observed to lead to infertility in several cases ^3^ . ROS is known to react with proteins, lipids, carbohydrates and nucleic acids, causing impaired cell function, DNA damage and apoptosis. The extent of testicular tissue damage is related to both the degree of twisting and the rapidity of surgical intervention to counter‐rotate both the testis and spermatic cord to re‐establish normal blood flow to the organ ^4^ . Previous studies using a rat model of testicular torsion have demonstrated that a 1-hour, 720° rotation of the testes followed by reperfusion results in permanent loss of spermatogenesis despite the return of blood flow. If treated within 6 hours of presenting with pain, there is a good chance of saving the affected testicle, as 90%-100% testicles will be saved. If treated within 6-12 h, 20%-50% testicles will be saved depending on the degree of the torsion, and if treated within 12-24 h, 0%-10% testicles will be saved ^5^ . Delayed surgery may result in orchiectomy or diminished fertility, and even if the testis is detorsed in time, infertility risk is the most worrisome complication ^6^ .

*Ganoderma lucidum (G. lucidum)* is a mushroom belonging to the Polyporaceae family of Basidiomycota and has been widely used as a traditional medicine for thousands of years, particularly in Asian countries ^7^ . The fruiting bodies, cultured mycelia and spores of *G. lucidum* contain a variety of bioactive chemical substances such as polysaccharides, triterpenoids and proteins ^8^ . Different experimental studies and modern clinical trials suggest that these active compounds isolated from *G. lucidum* have anti-inflammatory, antioxidant, antitumor and immunomodulatory activities ^9^ .

Though several compounds have been used to heal ischemia-reperfusion (I/R) injury in animal models of testicular torsion, few are available for use ^10^ . However, to date, there is no study on the effect of *G. lucidum* on a rat testicular torsion model. In our study, biochemical assays of malondialdehyde (MDA), superoxide dismutase (SOD), catalase (CAT), glutathione (GSH) levels, histopathology and expression levels of vascular endothelial growth factor (VEGF) and B-cell lymphoma 2 (Bcl-2) were examined in testicular tissue by immunohistochemical methods.

Thus, the aim of the present study was to determine whether treatment with *G.lucidum* with antioxidant properties ameliorated testicular damage caused by torsion/detorsion (T/D) injury using histopathological, biochemical and immunohistochemical methods.

## Methods

All experimental protocols conducted on animals were consistent with the National Institutes of Health Guidelines for the Care and Use of Laboratory Animals and were approved by the Health Sciences University, Ankara Education and Research Hospital Ethics Committee of Animal Care and Usage. Thirty male Wistar albino rats with a mean weight of 200-250 gr were used. They were housed in an air-conditioned room with 12-h light and dark cycles, where the temperature (23±2°C) and relative humidity (65–70%) were kept constant.

In this study, all surgical procedures were performed by the intramuscular injection of ketamine hydroxide (Ketalar ^®^ , Pfizer, Turkey) (50 mg/kg) and xylazine (Rompun ^®^ , Bayer, Germany) (10 mg/kg) for general anesthesia. All operations were performed under sterile conditions. Animals were randomly divided into three groups. Ten adult male rats were used in each group. Group 1 was assigned as the sham group. No treatment was applied to this group, and only the left testis was taken, and the rats were sacrificed under anesthesia. In Group 2 (T/D group), torsion was created by rotating the left testis 720° degrees clockwise along the longitudinal axis of spermatic cord for 2.5 hours under anesthesia. At the end of the 2.5 hours, the testis was corrected to its original position and left for 7 days for the detorsion procedure. In Group 3 (T/D+ *G. lucidum* ), after T/D, the rats were given *G. lucidum* polysaccharides (GLPS) (20 ml/kg) via gastric gavage for 7 days. Group 2 received similar volumes of the same fluid that did not contain G. *lucidum* polysaccharides. After 7 days of reperfusion, re-explorations and orchiectomies were performed on each rat in groups 2 and 3.

Testicular samples were fixed in neutral buffered formalin solution, directly dehydrated in ascending series of ethanol solution and embedded in paraffin wax. Five-micrometer sections were cut with a microtome (RM2265 rotary microtome; Leica, Germany) and mounted on coated slides. The sections were stained with Periodic Acid Schiff (PAS) and analyzed under a light microscope (Zeiss, Germany).

In this study, primary outcome measure consisted of the changes of MDA, SOD, CAT and GSH levels of biochemical parameters. Secondary outcome measure consisted of histopathologic and immunohistochemical changes.

### *Surgical procedure*

The rats were anesthetized by an intramuscular injection of ketamine hydroxide (50 mg/ kg) and xylazine (10 mg/ kg) under aseptic conditions. The tunica vaginal was removed with the help of a forceps to allow the testicular tissue to be visible. A scrotal pocket was created to place the testicle back into the scrotum after torsion. To create torsion, the left testis was rotated at 720°degrees clockwise around the longitudinal axis of spermatic cord for 2.5 hours. To prevent detorsion, the testis was fixed into the scrotal pocket by passing 4/0 non-traumatic absorbable suture through the dartos and testicular tunica albuginea. After 2.5 hours of torsion, the suture that fixed testis to dartos was cut for detorsion and the reperfusion period of the following 7 days.

### *Biochemical analysis*

MDA, SOD, CAT and GSH levels were examined in testicular tissue. Tissue samples were homogenized with ice-cold 150 mMKC. MDA levels were assayed for products of lipid peroxidation, and the results were expressed as nmol MDA/g tissue ^11^ . The SOD activity in the tissue was measured using the RANSOD kit (Randox Laboratories, Crumlin, UK). GSH was determined by the spectrophotometric method based on the use of Ellman’s reagent, and the results were expressed as μmol glutathione/g tissue ^12^ . CAT activity was determined by the spectrophotometric method based on the ability of hydrogen peroxide to form a stable stained complex with molybdenum salts ^13^ .

### *Immunohistochemical technique*

Formaldehyde-fixed testis tissue was embedded in paraffin wax for further immunohistochemical examination. Sections were deparaffinized in xylene. The antigen retrieval process was performed twice in citrate buffer solution (pH 6.0), first for 7 minutes, and then for 5 minutes, and was boiled in a microwave oven at 700 W. They were then allowed to cool to room temperature for 30 minutes and washed twice in distilled water for 5 minutes. Endogenous peroxidase activity was blocked in 0.1% hydrogen peroxide for 20 minutes. Ultra V block (Cat. No. 85-9043, Invitrogen, Carlsbad, California, USA) was applied for 10 minutes prior to the application of primary antibodies for Bcl-2 (cat: PA5-20068, Invitrogen, Carlsbad, California, USA) and VEGF (cat: PA5-16754, Invitrogen, Carlsbad, California, USA). Secondary antibody (Cat. No. 85-9043, Invitrogen, Carlsbad, California, USA) was applied for 20 minutes. Slides were then exposed to streptavidin-peroxidase for 20 minutes. Chromogen diaminobenzidine (DAB, Cat. No. 34002, Invitrogen, Carlsbad, California, USA) was used. Control slides were prepared as mentioned above, but by omitting the primary antibodies. After counter staining with hematoxylin and washing in tap water for 8 minutes and in distilled water for 10 minutes, sections were examined using a light microscope (Zeiss, Germany).

### *Statistical analysis*

Statistical analyses were conducted using SPSS (Version 22.0, SPSS Inc., Chicago, IL, USA). Descriptive statistics are presented as median (min-max) and mean ± standard deviation (SD). The normal distribution of the data was evaluated by Shapiro-Wilks test. Since data normality distribution was met, the groups were compared by an analysis of variance (ANOVA) followed by Tukey’s multiple comparison tests to determine which groups differed with pairwise comparison. The priori sample size and post-hoc power analyses were calculated using the G-power (Version 3.1) package. The sample size was calculated for ANOVA test, which was used to test the main hypothesis of the study. As a result of the sample size analysis performed using previous study knowledge, it was found that 30 rats, 10 in three different groups, needed to be involved in the study to reveal the significant differences in the groups using 80% power (1-β=0.80), α=0.05 error (95% confidence interval) with a two-sided hypothesis. Values of p<0.05 were considered statistically significant.

## Results

### *Biochemical results*

**Figure 1:**
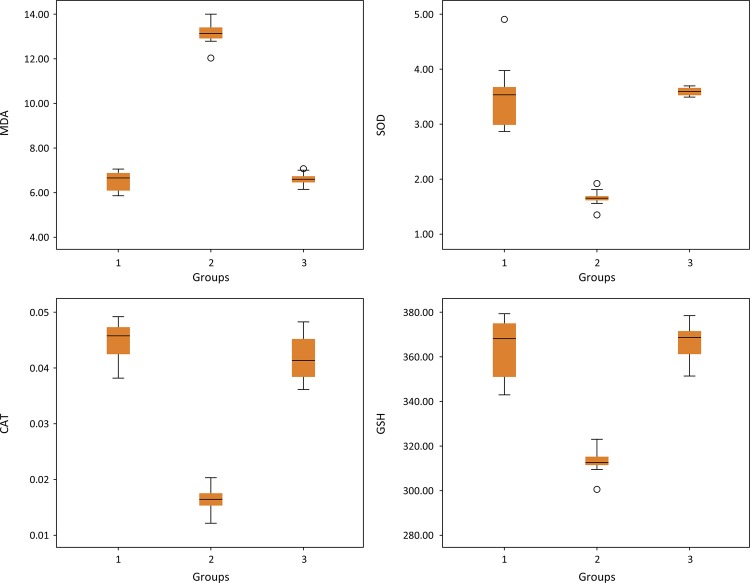
Comparison of MDA, SOD, CAT and GSH activities in control, T/D and T/D + *G. lucidum* groups on the boxplot graph. Groups; 1: Control; 2: Torsion-Detorsion; 3: Torsion-Detorsion+ *G. lucidum* MDA: malondialdehyde SOD: superoxide dismutase CAT: catalase GSH: glutathione

MDA levels in the testicular tissues of rats in the T/D group were significantly increased compared to the sham group, while the MDA levels in the T/D+ *G. lucidum* group were significantly decreased compared to the T/D group. *G. lucidum* treatment prevented the T/D-induced augmentation of MDA levels in the testis. The SOD, CAT and GSH activities of testicular tissue significantly decreased in the T/D group compared to the sham group. These values were significantly increased in the T/D+ *G. lucidum* group compared to the T/D group. The MDA, SOD, CAT and GSH levels of the sham, T/D and T/D + *G. lucidum* groups are shown in Table 1 ( Fig. 1 ).

**Table 1:** Comparison of MDA, SOD, CAT and GSH activities in groups 1, 2 and 3.

	Groups	N	Mean±SD	*P* value	Post-hoc P value
MDA	1	10	6.47±0.44	<0.001*	1-2: <0.001*
	2	10	13.08±0.52		1-3: 0.965
	3	10	6.52±0.29		2-3: <0.001*
SOD	1	10	3.52±0.62	<0.001*	1-2: <0.001*
	2	10	1.66±0.14		1-3: 0.861
	3	10	3.60±0.07		2-3: <0.001*
CAT	1	10	0.045±0.004	<0.001*	1-2: <0.001*
	2	10	0.016±0.002		1-3: 0.182
	3	10	0.042±0.004		2-3: <0.001*
GSH	1	10	363.1±12.5	<0.001*	1-2: <0.001*
	2	10	311.7±5.6		1-3: 0.822
	3	10	365.5±7.9		2-3: <0.001*

Statistically significant: p<0.05*

Groups; 1: Sham 2: Torsion-Detorsion 3: Torsion-Detorsion+ *G. lucidum*

MDA: malondialdehyde SOD: superoxide dismutase CAT: catalase GSH: glutathione SD: Standard deviation

Power of MDA: 100%, Power of SOD: 81%, Power of CAT: 100%, Power of GSH: 99%

### *Histopathologic results*

**Figure 2:**
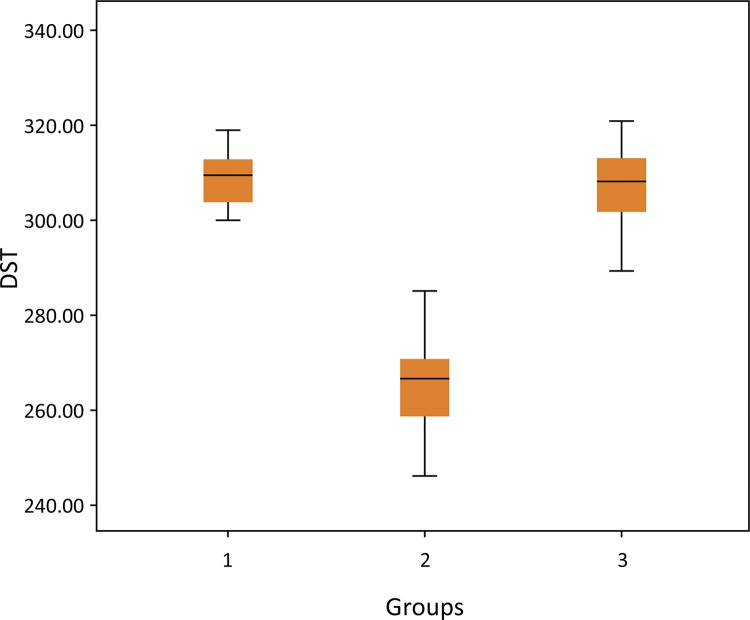
The boxplot graph of Diameter of Seminiferous Tubules (DST). Groups; 1:Control 2:Torsion-Detorsion 3:Torsion-Detorsion+ *G. lucidum*

Testicular tubule diameter measurements were evaluated among the groups. There was a significant difference between the sham, T/D and T/D+ *G.lucidum* groups (p<0.001). According to the tube diameter measurement results, a significant improvement in the tube diameter was observed in the group treated with *G.lucidum* after T/D injury ( Table 2 , Fig. 2 ).

**Table 2:** Diameter of seminiferous tubules.

	Groups	N	Mean±SD	*P* value	Post-hoc *P* value
Diameterof Semiferous tubules	1	10	308.9±6.22	<0.001*	1-2: <0.001*
	2	10	265.3±12.27		1-3: 0.930
	3	10	307.33±9.04		2-3: <0.001*

Groups; 1: Sham 2: Torsion-Detorsion 3: Torsion-Detorsion+ *G. lucidum*

Power 100%

**Figure 3:**
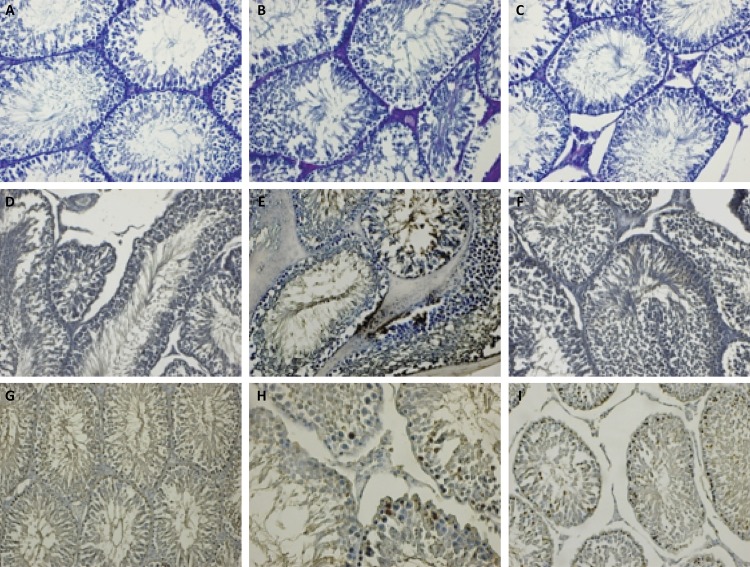
A: Sham group: The spermatogenic cells of the seminiferous tubules in the control group with strong mitotic activity, Sertoli cells with regular broad luminal faces. Normal membrane thickness, interstitial area, blood vessels and Leydig cells, PAS staining Bar 50µm. B: T/D group: An increased thickness of basal membrane in tubules, pyknosis and deterioration in the nucleus of spermatogenic cells, lack of maturation in sperm cells and degeneration of Sertoli cells, hemorrhage and dilatation of blood vessels, an increase in connective tissue, degeneration of Leydig cells, PAS staining Bar 50µm. C: T/D+G. lucidum : Decreased basal membrane thickness of tubules, degeneration and deterioration of spermatic cells in some tubules and mitotic increase of spermatic cells in many tubules, small hemorrhage in interstitial blood vessels, heterochromatin appearance of Leydig cells, PAS staining Bar 50µm. D: Sham group: Negative VEGF expression in spermatogenetic and Sertoli cells in tubules, interstitial vascular endothelial cells and Leydig cells, VEGF staining Bar 50µm. E: T/D group: Positive expression of VEGF on apical surfaces of spermatogenic cells and Sertoli cells in tubules, VEGF expression positive in endothelial cells and some inflammatory cells in interstitial blood vessels and also in Leydig cells, VEGF staining Bar 50µm. F: T/D + G. *lucidum* group: positive VEGF expression in a few spermatogenic cells in some tubules, especially in vascular endothelial cells, Sertoli cells and negative VEGF expression in Leydig cells, VEGF staining Bar 50µm. G: Sham group: Negative Bcl-2 expression in spermatogenic and Sertoli cells in the basal part of the tubules, positive Bcl-2 expression in some small spermatid cells near the lumen, Bcl-2 staining Bar 100µm. H: T/D group: Dense Bcl-2 expression in spermatogenic cells showing potent mitotic activity in basal layers of tubules, Bcl-2 expression on the luminal faces of Sertoli cells, Bcl-2 staining Bar 50µm. I: T/D +G. *lucidum* group: Bcl-2 positive reaction in several spermatogenic cells in basal and apical regions of tubules, negative Bcl-2 reaction in Sertoli and Leydig cells, Bcl-2 staining Bar 50µm.

Testicular tissue was histologically examined under light microscope for all groups. In the sham group, seminiferous tubules had normal structure. Spermatic cells in seminiferous tubules were regular oval and mitotically active towards the lumen. The large luminal faces of Sertoli cells were regularly seen. The membrane thickness was normal. The intertubular space, connective tissue cells, blood vessels and Leydig cells were normal ( Fig. 3A ). In the T/D group, basal membrane thickness of tubules increased. Pyknosis and deteriorated nuclei of the spermatogenic cells, attenuated maturation in the sperm cells and degenerated Sertoli cells were observed. Hemorrhage and dilatation in the blood vessels of the interstitial area, increased connective tissue and degenerated Leydig cells were prominent ( Fig. 3B ). The T/D+ *G. lucidum* group showed a decrease in the basal membrane thickness of tubules compared to the T/D group. Degenerated and deteriorated spermatic cells were evident in some tubules, along with mitotic increase. Little hemorrhage was observed in the blood vessels of the interstitial regular connective tissue cells and heterochromatin Leydig cells ( Fig. 3C ).

### *Immunohistochemical results*

In the sham group, negative VEGF expression was observed in germ and Sertoli cells, vascular interstitial endothelial cells and Leydig cells ( Fig. 3D ). In the T/D group, the expression of VEGF in the apical faces of spermatogenic cells and Sertoli cells was positive. VEGF expression was positive in endothelial, some inflammatory, Leydig cells and interstitial blood vessels ( Fig.3E ). In the T/D+ *G. lucidum* group, positive VEGF expression was observed in a small number of spermatogenic cells, particularly Sertoli cells. However, it was negative in interstitial endothelial cells and Leydig cells ( Fig. 3F ).

Bcl-2 expression was negative in spermatogenic cells and Sertoli cells in the sham group, while it was positive in several small spermatid cells near the lumen ( Fig. 3G ). In the T/D group, intense Bcl-2 expression was observed in spermatogenic cells with strong mitotic activity in the basal layers of tubules. Significant expression of Bcl-2 was observed in the luminal faces of some Sertoli cells ( Fig. 3H ). In the basal and apical regions of the tubules, Bcl-2 reaction was positive in a few spermatogenic cells, whereas it was negative in Sertoli and Leydig cells. Bcl-2-positive germinal cells were also fewer in the *G. lucidum* -treated group compared to the T/D group ( Fig. 3I ).

## Discussion

In our study, MDA levels were found to be significantly higher in the T/D group compared to the Sham and T/D+ *G. lucidum* groups. The SOD, CAT and GSH levels were found to be significantly lower in the T/D group compared to the Sham and T/D+ *G. lucidum* groups. There was a decrease in the basal membrane thickness of tubules in the T/D+ *G. lucidum* group compared to the T/D group. While the T/D group was observed to have erupted spermatogenic cells with impaired nuclei, weakened maturation in the sperm cells and degenerated Sertoli cells, degenerated and impaired spermatic cells and mitotic increase were evident in some tubules of the T/D+ *G. lucidum* group. Bleeding and dilatation of blood vessels in the interstitial region, increased connective tissue and impaired Leydig cells were evident in the T/D group, whereas a small amount of bleeding was observed in the blood vessels of the interstitial regular connective tissue cells and the heterochromatin Leydig cells in the T/D+ *G. lucidum* group.

In the present study, we did not study the effect of *G. lucidum* on testicular I/R injury at different doses or different administration times. Detailed randomized controlled prospective clinical studies are required to assess the efficacy of *G. lucidum* on testis torsion.

Ozbek *et al* . ^14^ suggested in an experimental study that testicular torsion significantly decreased SOD and CAT levels when compared to the control groups. In our study, the SOD and CAT activities in testicular tissue significantly increased in the T/D+ *G. lucidum* group compared to the T/D group. Mestrovic et al. suggested that GSH activities in the ipsilateral testes of the treatment group were significantly higher than those in the T/D group ^15^ . In our study, GSH activities in testicular tissue significantly increased in the T/D+ *G. lucidum* group compared to the T/D group. Results from I/R studies of *G. lucidum* in brain, kidney and heart comply with the results of our study with decreased MDA and increased SOD and GSH ^16^ . In the literature, there are testis I/R studies using apocynin, nifedipine and urapidil. The results of these studies comply with ours with these medications reducing MDA levels and increasing SOD and GSH, showing antioxidant effect ^14 , 15 , 17^ . Histological alterations similar to the results in our study were also demonstrated in another testicular I/R study conducted by Ozbek *et al* . ^14^ . Hirai et al. reported the protective effect of VEGF on histological damage in testicular torsion by preserving spermatogenic activity ^18^ . Tunçkıran *et al* . ^19^ suggested that administering VEGF before reperfusion might have the potential to decrease long-term histologic damage after testicular torsion. In our study, VEGF expression was positive in the T/D group in the endothelial cells in the interstitial blood vessels, in some inflammatory cells and Leydig cells, in the apical faces of the spermatogenic cells and in Sertoli cells. In the T/D+ *G. lucidum* group, positive VEGF expression was observed in a small number of spermatogenic cells, especially in Sertoli cells, and it was negative in interstitial endothelial cells and Leydig cells. This may indicate that *G. lucidum* has a protective effect on VEGF expression linked to the testis. Kanter *et al* . ^12^ stated that sensitivity to modulated apoptotic factors was higher in spermatogonia and spermatocytes, whereas Sertoli and Leydig cells were highly resistant. A study showed that the amount of VEGF and Bcl-2 both decreased in testicular tissue, showing positive correlation in diabetic rats, and that both proteins were involved in cell proliferation, apoptosis and angiogenesis in the pathophysiology of diabetes ^13^ . In our study, intense Bcl-2 expression was observed in spermatogenic cells with strong mitotic activity in the basal layers of tubules in the T/D group. Bcl-2 expression was also found to be higher in Sertoli cells. Bcl-2-positive germinal cells were fewer in the *G. lucidum* -treated group compared to the T/D group. G.lucidum administered after T/D injury was estimated to decrease the effect of Bcl-2 expression and inhibit apoptosis. A similar study by Sumii *et al* . ^20^ showed that urocortin reduced Bcl-2 expression and had an antiapoptotic effect.

## Conclusions

*G. lucidum* administration may decrease oxidative stress and histopathological damage. *G. lucidum* has a significant protective effect against testicular T/D damage in rats. This protective effect is thought to be mainly due to its antioxidative property, and it is thought to induce angiogenic effect and decrease apoptotic development. The use of *G. lucidum* therapy after reperfusion may be an alternative to germ cell degeneration resulting from testicular torsion and associated infertility.
